# Obituary

**Published:** 1848-02

**Authors:** 


OBITUARY.

*Death of Dieffenbach and Liston.*—Surgery has recently lost two of its brightest luminaries in the death of these distinguished men. Dieffenbach died very suddenly, apparently in good health. It is stated in one of our exchange journals that, "on the morning of the 11th inst., (November,) M. Dieffenbach arrived at the Hospital of La Charité at Berlin, in his usual state of health, and, before commencing his clinical lecture, he was visited by two French physicians. He introduced them to a patient on whom he had operated for aneurism the day before, and he then sat down on the sofa with his two visiters, and described to them the steps of the operation. It was observed that he suddenly became silent, and inclined his head towards one of these gentlemen, as if about to whisper to him. As he did not move from his position, it was suspected that he was ill. Dr. Angelstein was immediately on the spot, and attempted to bleed him, but it was found that life had ceased. No inspection had been made, and the cause of death was unknown. M. Dieffenbach was born at Konigsberg in 1795, and was consequently in the fifty-second year of his age." His remains were deposited on the 15th of November, in the Werder

Cemetery, without the gates of Berlin, and amid the uncontrollable expressions of regret of a host of citizens of all classes. In its progress to the grave, quantities of flowers and coronets of "immortelles" were showered on the coffin by the people, and every demonstration of sorrow suited to the occasion was exhibited by the people and the Sovereign.

Robert Liston was born on the 28th of October, 1794, in the parish of Ecclesmachan, Linlithgow, West Lothian, Scotland, and died in London on the 7th of December, 1847, in the fifty-third year of his age. He was a man of robust form, and most active in his habits. He had reached almost the highest honours his profession could bestow, when seized with the illness which terminated his life.

"At the beginning of the year (1847) he had complained of difficulty of swallowing; but this excited little uneasiness, till last July, when he was suddenly seized with hæmorrhage from the throat, by which he lost above thirty ounces of blood. The source of this hæmorrhage was not ascertained; and by a little care for a few weeks he recovered his health sufficiently to resume his practice. On the first of December he had an attack of difficulty of breathing, which continued with little or no relief till the 7th, when he died."

"The thorax was examined, thirty-six hours after death, by Mr. Cadge, in the presence of Sir. B. Brodie, Drs. Watson, Latham, and Forbes, and Mr. J. Dalrymple. The lungs were found but slightly collapsed, congested throughout, but otherwise perfectly healthy; the pericardium contained about an ounce of transparent yellowish serum; the heart itself was healthy, saving a slight atheromatous deposit in the mitral and aortic semilunar valves. On removing the subclavian vein and cellular tissue from the arch of the aorta, the cause of death became at once apparent. An aneurism as large as an orange, flattened from before backwards, was seen pressing back the trachea; it arose from the upper part of the arch, close behind the left carotid artery, at the origin of the innominata, which seemed almost to commence from the aneurismal pouch; the communication with the aorta was by a circular opening, as large as a half-crown. On opening the trachea from behind, the mucous membrane was seen to be very dark and congested, and in its front part, where it was firmly connected to the tumour, there were three or four whitish prominences, as large as split peas, situated between the rings; it was at first difficult to understand what these elevations really were, but on slitting up the pouch and removing the fibrinous laminæ, they were drawn from between the ring, leaving the latter quite bare, and the trachea perforated in three or four points; they were, in short, portions of the clot, which half filled the sac of the aneurism. The source of the hæmorrhage and the cause of death were at once explained.—*Lancet*.

A brief but interesting account of these eminent men, is contained in an article in our Record, extracted from the London and Edinburgh Journal.

